# Brachyury, Foxa2 and the *cis*-Regulatory Origins of the Notochord

**DOI:** 10.1371/journal.pgen.1005730

**Published:** 2015-12-18

**Authors:** Diana S. José-Edwards, Izumi Oda-Ishii, Jamie E. Kugler, Yale J. Passamaneck, Lavanya Katikala, Yutaka Nibu, Anna Di Gregorio

**Affiliations:** Department of Cell and Developmental Biology, Weill Medical College of Cornell University, New York, New York, United States of America; Harvard University, UNITED STATES

## Abstract

A main challenge of modern biology is to understand how specific constellations of genes are activated to differentiate cells and give rise to distinct tissues. This study focuses on elucidating how gene expression is initiated in the notochord, an axial structure that provides support and patterning signals to embryos of humans and all other chordates. Although numerous notochord genes have been identified, the regulatory DNAs that orchestrate development and propel evolution of this structure by eliciting notochord gene expression remain mostly uncharted, and the information on their configuration and recurrence is still quite fragmentary. Here we used the simple chordate *Ciona* for a systematic analysis of notochord *cis*-regulatory modules (CRMs), and investigated their composition, architectural constraints, predictive ability and evolutionary conservation. We found that most *Ciona* notochord CRMs relied upon variable combinations of binding sites for the transcription factors Brachyury and/or Foxa2, which can act either synergistically or independently from one another. Notably, one of these CRMs contains a Brachyury binding site juxtaposed to an (AC) microsatellite, an unusual arrangement also found in Brachyury-bound regulatory regions in mouse. In contrast, different subsets of CRMs relied upon binding sites for transcription factors of widely diverse families. Surprisingly, we found that neither intra-genomic nor interspecific conservation of binding sites were reliably predictive hallmarks of notochord CRMs. We propose that rather than obeying a rigid sequence-based *cis*-regulatory code, most notochord CRMs are rather unique. Yet, this study uncovered essential elements recurrently used by divergent chordates as basic building blocks for notochord CRMs.

## Author Summary

Transcription factors control the spatial and temporal expression of a multitude of genes by binding their *cis*-regulatory modules (CRMs). In this study, we investigated the architecture and composition of CRMs that direct gene expression in the notochord, a structure necessary for the support and patterning of the embryonic body plan of all chordates. We used the simple chordate *Ciona* to carry out a comparative study of notochord CRMs and we identified the sequences necessary for their function. These sequences, in turn, highlighted the existence of multiple mechanisms that enable gene expression in the notochord. Surprisingly, combinations of binding sites identical to those found in active CRMs were not necessarily able to direct notochord gene expression and were often poorly conserved among cogener species. These results challenge the concept of a notochord-specific *cis*-regulatory “code”, and outline the limitations of methods for CRM identification that rely upon interspecific conservation of non-coding sequences. Nevertheless, a broad comparison of the structure of the *Ciona* CRMs with that of the notochord CRMs characterized thus far from all chordates outlines the existence of essential evolutionarily conserved building blocks, such as binding sites for the transcription factors Brachyury and Foxa2, that are shared by subsets of these regulatory modules.

## Introduction

*Cis*-regulatory modules (CRMs), or enhancers, are genomic DNA regions that dictate location, timing and rate at which one or more genes are expressed [1]. These regions have variable length and contain a flexible number of binding sites for transcription factors that function as either activators or repressors [2]. Point mutations in one or more of the functional binding sites within a CRM can alter its spatial and temporal properties, or cause its partial or complete inactivation. Recent estimates suggest that the human genome contains hundreds of thousands of CRMs that are believed to be mainly responsible for the developmental and functional complexity of different cells, tissues, and organs [3]. Notably, mutations and deletions of human enhancers have been associated with developmental defects, disease, and cancer [4–6]. However, in the human genome, as well as in several others, CRMs can be located up to thousands of kilobases away from the genes that they control and are brought closer to their target promoters after being bound by specialized proteins that bend the DNA [7]. Furthermore, CRMs can be located within introns and/or other untranslated regions [8], or can be grouped into synergistically acting clusters called super-enhancers [9]. The crucial roles of CRMs, their complexity and their elusive nature, render a *cis*-regulatory code a highly desirable tool that would greatly simplify the genome-wide identification of CRMs with related properties. Studies aimed at identifying tissue-specific *cis*-regulatory codes have focused on genome-wide searches of clusters of known transcription factor binding sites [10] and on interspecific conservation of clusters of binding sites and/or larger non-coding sequences [11]. Nevertheless, recent research suggests that conserved clusters of binding sites are often non-functional [12] and that even evolutionarily ultraconserved genomic regions do not necessarily possess *cis*-regulatory activity [13].

The aim of the present study was to determine the structure and the functional binding sites of CRMs that shared comparable *cis*-regulatory activity and were presumably co-regulated, and to look for elements that could define a tissue-specific *cis*-regulatory code. We centered our analysis on CRMs active in the notochord, the most distinctive of chordate synapomorphies [14,15]. In all chordates, the notochord is the main source of support for the developing embryo and an essential patterning center for many of its structures and organs [16]. In vertebrates, the notochord is replaced by the vertebral column and its remnants form the *nuclei pulposi* of the intervertebral discs [17]. For the present study we used as a model system the tunicate *Ciona*, an invertebrate chordate that couples a compact, fully annotated genome with ease of transgenesis and tractable notochord [18,19]. According to phylogenomics data, tunicates are the invertebrate chordates most closely related to vertebrates [20], and thus provide an opportunity to reconstruct the genetic circuitry and the evolutionary origins of the notochord through the identification of *cis*-regulatory sequences that enable gene expression in this structure [21–23].

We began this analysis with the characterization of fourteen notochord CRMs from *Ciona*. After isolating the minimal sequences necessary for their function, we tested whether these minimal sequences could be used to predict related notochord CRMs. We also evaluated the evolutionary conservation of CRM sequences between two *Ciona* species, *C*. *intestinalis* and *C*. *savignyi*, and compared the structure of the *Ciona* notochord CRMs to fully characterized notochord CRMs from other chordates, including mouse and zebrafish.

Rather than a *sensu stricto cis*-regulatory code, this study elucidated various combinations of functional transcription factor binding sites that function in a context-dependent fashion. These binding sites are often poorly conserved interspecifically, and therefore would have been missed by conservation-based methods of enhancer detection. However, despite the intraspecific and interspecific variability in their composition and function, binding sites for Brachyury and Foxa2 emerged as recurrent hallmarks of notochord CRMs from highly divergent chordates.

## Results and Discussion

**Fig 1:**
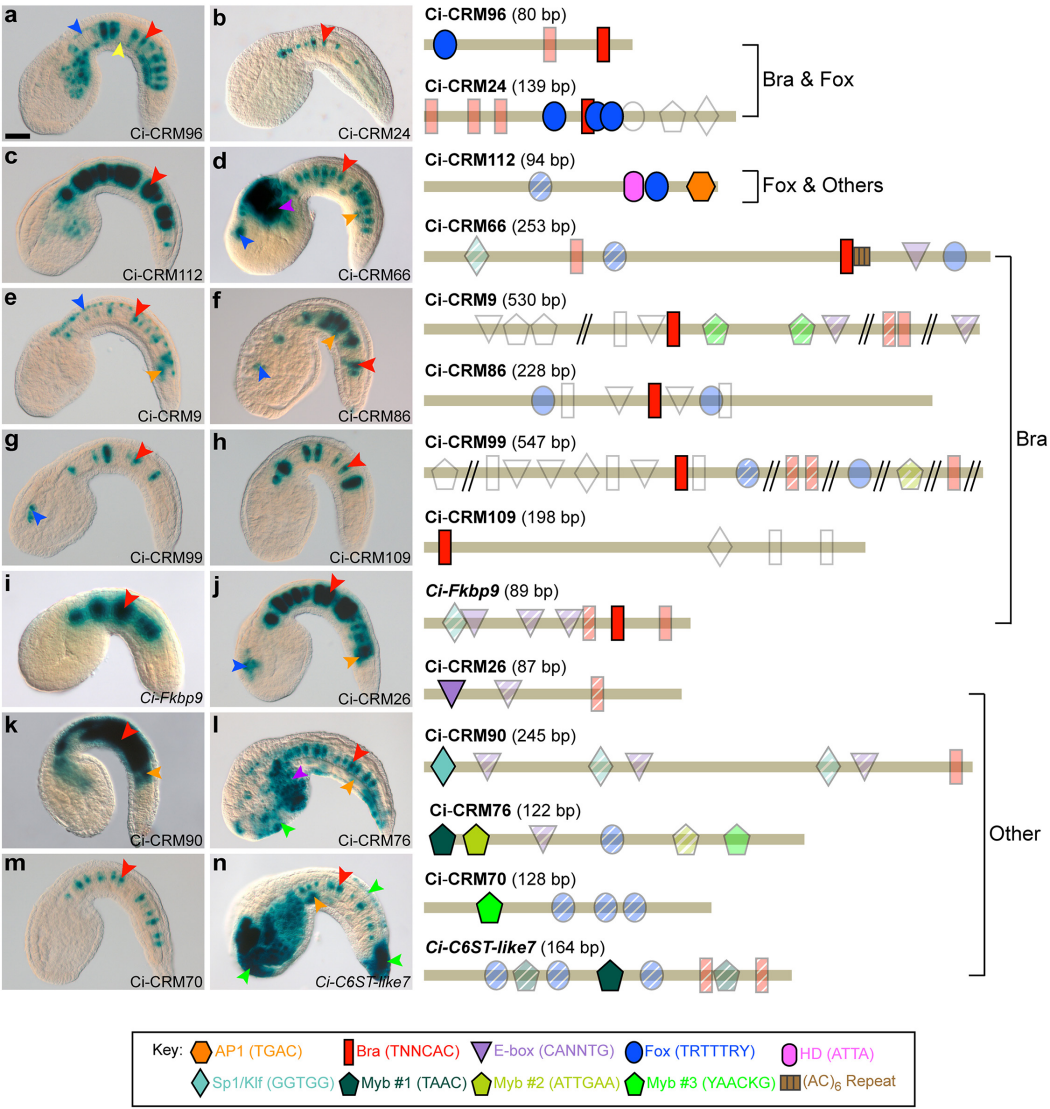
A comparative study of notochord CRMs in *Ciona*. **a-n:** Microphotographs of transgenic *Ciona* embryos expressing the *LacZ* reporter in the notochord (red arrowheads) under the control of 14 CRMs. (Right) Schematic representations of the 14 minimal notochord CRMs. Putative transcription factor binding sites are mapped along the length of each enhancer (tan bar), as indicated in the key (bottom). Point mutations uncovered site(s) required for notochord expression (colored and opaque) as well as sites that did not evidently contribute (colored, but transparent). Putative binding sites deemed dispensable through truncations are colored and hatched. Untested putative sites are outlined in gray. Additional staining domains are indicated by arrowheads, colored as follows: blue: CNS, yellow: endoderm, orange: muscle, purple: mesenchyme, green: epidermis. Embryos are oriented with dorsal up and anterior to the left. Scale bar: 40 μm. See also S1, S2 and S3 Figs

We identified fourteen CRMs that can induce gene expression in the *Ciona* notochord. To avoid sequence and/or positional biases, all but one of the notochord CRMs (Fig 1) were isolated through testing of random genomic regions (S1 Table). Minimal notochord enhancers spanning 80–547 bp were subsequently identified through sequence-unbiased truncation analyses, involving *in vivo* testing of ~200 constructs (S1, S2 and S3 Figs). Lastly, we assessed the effects of site-directed mutations targeting either known putative transcription factor (TF) binding sites or uncharacterized sequences. The results of these studies are condensed in Fig 1.

We found that the majority of the CRMs (9/14, 64.3%) require binding sites for the TFs *Ciona* Brachyury (Ci-Bra) and/or Ci-FoxA-a (Foxa2/fkh/HNF3beta ortholog; hereinafter Ci-Fox); in contrast, binding sites for TFs of widely different families were responsible for the function of the remaining five notochord CRMs. This analysis also revealed unexpected characteristics of these regulatory elements. For instance, enrichment for a particular binding site was not a reliable predictor of either functionality or cooperativity (*e*.*g*., all Ci-Fox sites in Ci-CRM70 are dispensable; Figs 1 and S1). In some instances, only one of the multiple copies/variants of a given TF binding site was required for notochord gene expression (*e*.*g*., only one of the seven Ci-Bra sites in Ci-CRM99 is necessary; Figs 1 and S3). Furthermore, even CRMs necessitating the same types of binding sites could function differently: a Myb-like site worked individually in one CRM (*Ci-C6ST-like7*), and in combination with a related Myb-like site in another (Ci-CRM76) (Figs 1 and S1).

**Fig 2:**
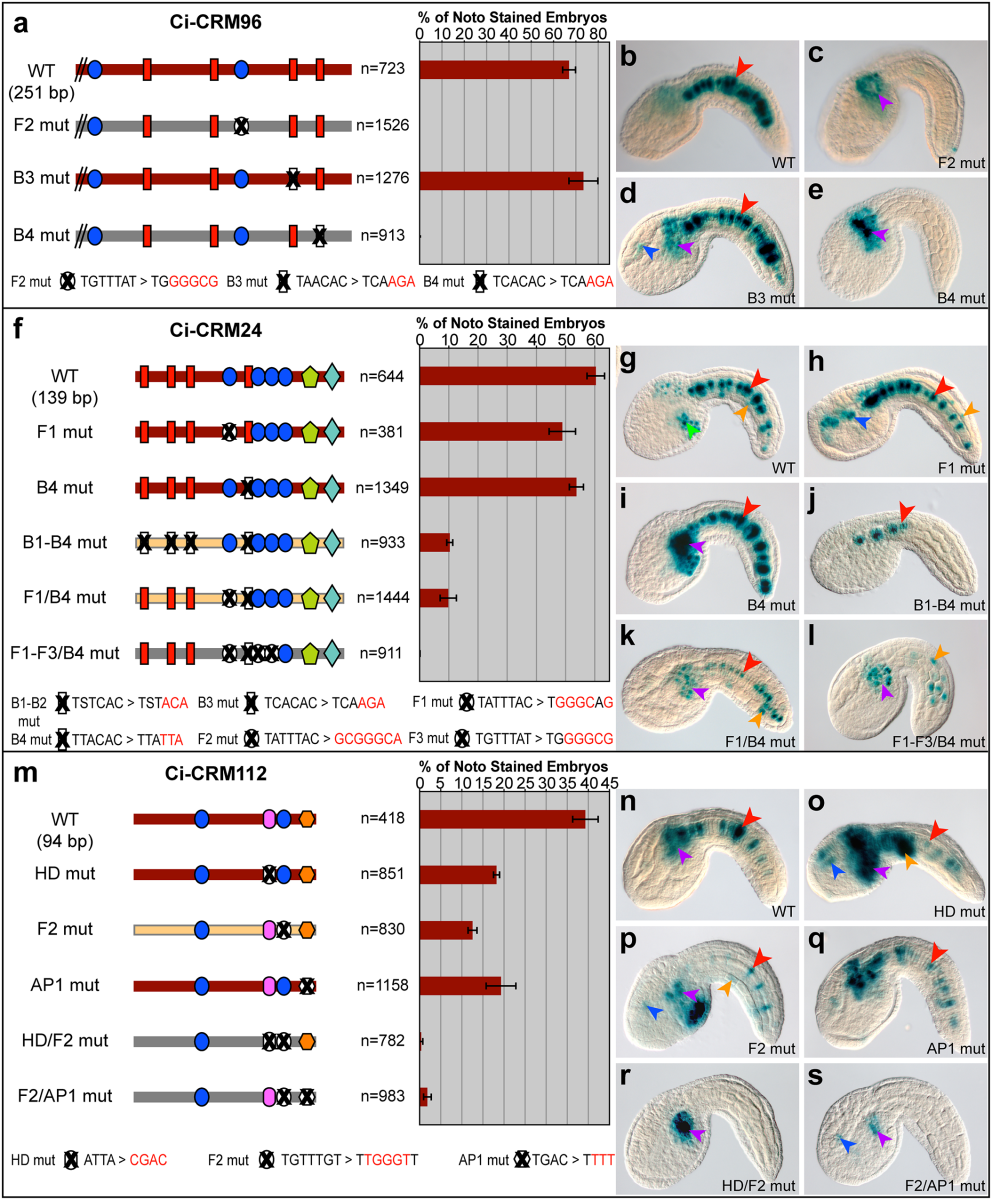
Alternative regulatory mechanisms of notochord CRMs requiring Ci-Bra and/or Ci-Fox binding sites. **a,f,m:** (Left) Schematic representations of wild-type (WT) and site-directed mutant CRMs; TF binding sites are as in Fig 1, with the mutant sequences indicated at the bottom of each panel. Mutated binding sites are colored in white and covered by “X” signs. Maroon bars represent constructs able to elicit notochord expression, while configurations exhibiting weak or no notochord staining are depicted by yellow and gray bars, respectively. (Right) Quantification of the fraction of the total stained embryos showing notochord expression after electroporation of the constructs at the left of each bar. n: number of fully developed stained embryos. Error bars denote standard deviation from the mean. **b-e, g-l, n-s:** Microphotographs of embryos expressing the transgenes indicated at the bottom right of each panel. Arrowheads are color-coded as in Fig 1. Abbreviations: WT: wild-type, F: Fox binding site, B: Brachyury binding site, HD: homeodomain, AP1: activator protein 1, mut: mutated, noto: notochord. In **f**, “S” stands for C/G. See also S2 Fig.

We had previously described a notochord CRM, associated with the gene *Ci-tune*, activated by synergistic Ci-Bra and Ci-Fox binding sites [24]. In this study, we found that Ci-CRM96 relies on the same type of synergism (Fig 2A), and although the sequences of the Ci-Bra and Ci-Fox sites differ between these two CRMs, their spacing is comparable (48 bp in Ci-CRM96, 46 bp in *Ci-tune*). In contrast, the multiple Ci-Bra and Ci-Fox sites in Ci-CRM24 act redundantly, as individual mutations (*e*.*g*., Fox1 and Bra4, Fig 2F) are not detrimental to notochord staining (Fig 2F–2I), and reduction/loss of notochord staining is only obtained through compound mutations (Fig 2F, 2J, 2K and 2L). Unlike the previous CRMs, Ci-CRM112 is devoid of Ci-Bra sites (Fig 2M). In this case, putative homeodomain (HD) and activator protein 1 (AP1) sites appear to work cooperatively with a Ci-Fox site, since all single mutations decrease notochord staining (Fig 2M–2Q), and simultaneous mutations of the functional Ci-Fox site and either the HD or AP1 sequences result in loss of staining (Figs 2M, 2R, 2S and S2).

**Fig 3:**
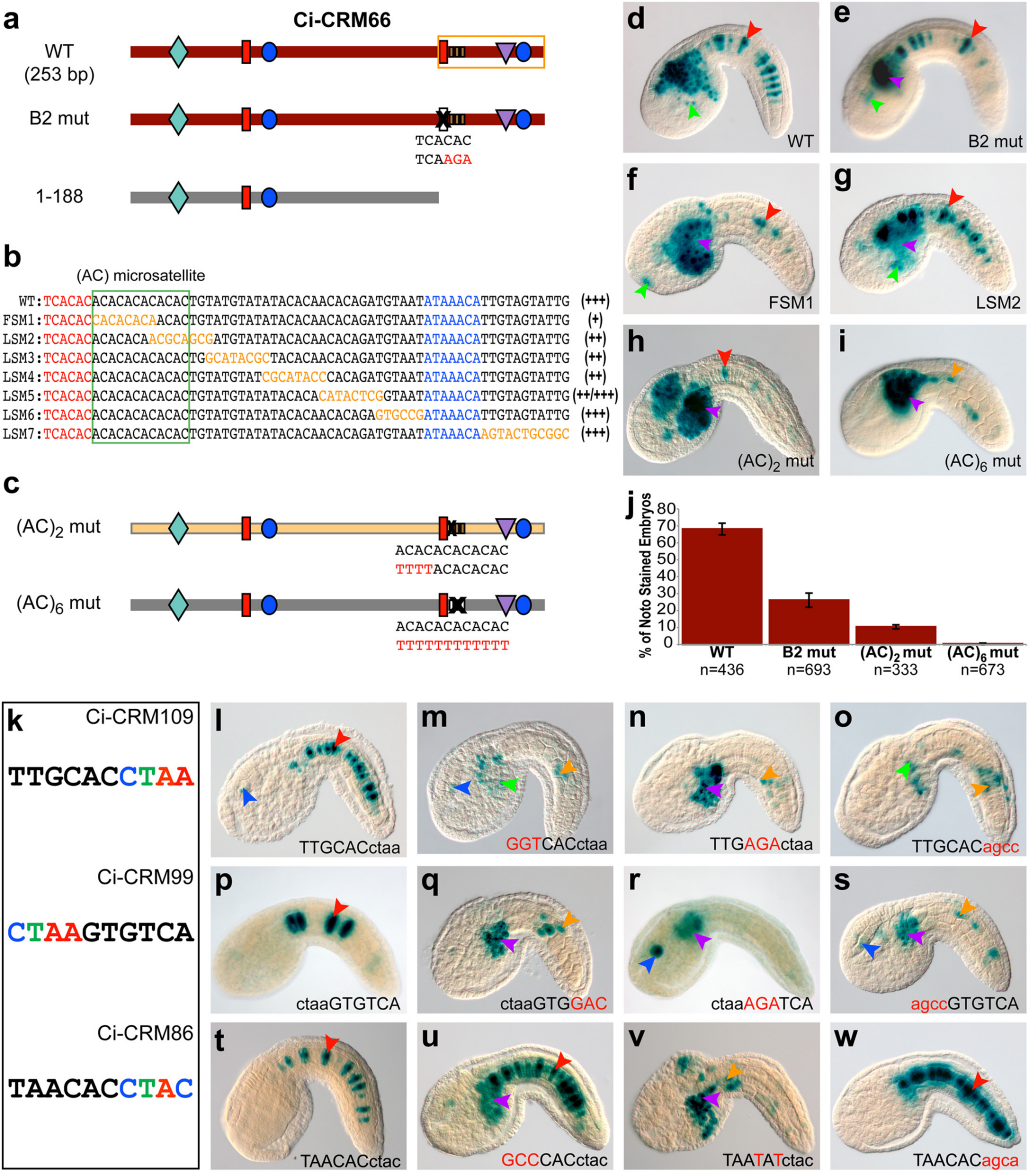
The function of individual Ci-Bra binding sites can be modulated by either an (AC) microsatellite or a flanking sequence. **a,c:** Schematic representations of wild-type (WT) and mutant CRMs, as described and colored in Fig 2; the (AC) microsatellite sequence is schematized as a segmented brown rectangle. **b:** Mutational series of the area boxed in orange in the 253-bp construct. Red and blue nucleotides correspond to the Ci-Bra and Ci-Fox sites, respectively, and orange nucleotides indicate the bases changed in each mutant plasmid. The (AC)_6_ microsatellite sequence is boxed in green. The relative ability of each construct to direct notochord gene expression is shown by plus signs at the right of each sequence. **d-i:** Photos of embryos electroporated with the constructs depicted in **a**,**b**,**c**; arrowheads are color-coded as in Fig 1. **j:** Quantification of notochord-stained embryos harboring the constructs in **a**,**c**. Error bars indicate standard deviation from the mean. **k:** Identification of an extended CTAM sequence (colored) shared by a subset of individually-acting Ci-Bra binding sites. **l-w:** Microphotographs of embryos carrying wild-type CRMs (**l,p,t**) compared to embryos carrying various mutant versions of Ci-CRM109 (**m-o**) Ci-CRM99 (**q-s**) and Ci-CRM86 (**u-w**). Core Ci-Bra binding sites are capitalized. Mutations are depicted in red. Abbreviations: FSM: “frame-shift” mutation, LSM: linker scanning mutation. See also S3 Fig.

Six CRMs rely on individual Ci-Bra binding sites (Figs 1, S1 and S3). Counterintuitively, the sequences of indispensable Ci-Bra sites differ for each Ci-Bra-dependent CRM, and sites with identical core sequences may be necessary in one context, but not in another (*e*.*g*., the TTGCAC sites in Ci-CRM109 and *Ci-Fkbp9*; S1 and S3 Figs). To uncover the molecular foundations of such differences, we assessed the roles of sequences directly adjacent to the necessary Ci-Bra binding sites. For Ci-CRM66, which lies within an intron of *Ci-Ephrin3*, we found that mutation of a single Ci-Bra binding site drastically decreased, but did not abolish, notochord staining (Figs 3A, 3E, 3J and S3). Linker-scanning mutagenesis revealed that the most detrimental mutations were those affecting an (AC)_6_ microsatellite [25] directly abutting the TCACAC Ci-Bra site (Fig 3B). Mutation of the first two (AC) pairs (Fig 3C) caused a sharp drop in notochord expression (Fig 3H and 3J), as did a mutation that caused a “frame-shift” of the microsatellite sequence (Fig 3B and 3F), suggesting that uninterrupted periodicity between the Ci-Bra binding site and this sequence may be required for the function of this CRM. The number of intact repeats also influenced activity (Fig 3B), and the mutation of the entire microsatellite abolished notochord expression (Fig 3C, 3I and 3J). Notably, ChIP-chip studies of genomic targets of Brachyury in differentiating mouse embryonic stem cells showed that this TF often binds (AC) repeats [26]. The *Ciona intestinalis* genome contains only nine copies of an (AC)_≥6_ microsatellite abutting a TCACAC Ci-Bra binding site; however, despite their reported occupancy by Ci-Bra in early embryos [27], none of the remaining eight regions directed notochord gene expression (S2 Table).

We also searched the sequences of the remaining five CRMs that rely on single Ci-Bra binding sites for clues on the mechanisms that might create the appropriate context for their function. Even though mouse Brachyury was initially found to bind the palindromic sequence T(G/C)ACACCTAGGTGTGA [28], it was later shown that TNNCAC core half-sites are efficiently bound by Brachyury proteins from mouse and other organisms, including *Ciona* [29–32]. Our results confirm that a palindromic organization is not required; instead, we observed that 50% of the required Ci-Bra sites matched either the TNNCACCTAM or the CTAMGTGNNA consensus (core sites underlined) (Fig 3K). Consequently, we selectively mutated the adjacent nucleotides while leaving the TNNCAC cores intact and found that in the case of Ci-CRM109 and Ci-CRM99 disruption of the CTAM sequence had the same effect as the mutation of the cores (Fig 3L–3S). Similar results were obtained through the mutation of this stretch in the *Ci-ABCC10* CRM [33]. In contrast, mutation of the CTAM sequence within Ci-CRM86 left notochord staining unaffected (Fig 3T–3W) and a CTAM-containing Ci-Bra binding site within Ci-CRM9 was found to be dispensable (S3 Fig). We conclude that the CTAM extension is not entirely predictive of whether a CRM will necessitate a single Ci-Bra site, and the binding sites that possess it are not always necessary. It is also conceivable that a fraction of the binding sites that we tentatively attributed to Ci-Bra might be interchangeably or exclusively utilized by Ci-Tbx2/3, the only other T-box protein present in the *Ciona* notochord, which acts as a mediator of Ci-Bra [34]. The sequences flanking the core TNNCAC site might therefore be required for binding specificity of either T-box factor, Ci-Bra or Ci-Tbx2/3.

In the last group of five minimal CRMs, the sequences required for notochord expression were neither Ci-Bra nor Ci-Fox binding sites (Fig 1), but instead resembled sites for bHLH (Ci-CRM26), Klf/Sp1 (Ci-CRM90), and Myb-like factors (Ci-CRM70, Ci-CRM76 and *Ci-C6ST-like7*) (S1 Fig). These results are consistent with previous reports of notochord-expressed bHLH, Klf6 and Klf15 TFs [35–37], and of a *Myb*-related gene in *Ciona* [38]. The requirement for two short Myb-like sites in Ci-CRM76 (Fig 1) led us to hypothesize that its activity might require a specific architecture. Accordingly, we found that while reversing the orientation of one of the Myb-like sites (abbreviated as “M”), M2-2, had no effect, transposing the order of the two required Myb-like sites, M1-5 and M2-2, largely decreased notochord staining (S4 Fig). Furthermore, increasing the spacing between M1-5 and M2-2 (4 bp) to that of the dispensable sites, M2-1 and M1-4 (8 bp), caused an even more substantial reduction of reporter gene expression in the notochord (S4 Fig). Nevertheless, seven genomic regions containing Myb-like sites with the identical composition, orientation and spacing as Ci-CRM76, all of which mapped near notochord genes, did not yield detectable notochord expression when tested *in vivo* (S3 Table).

Additional sequence inspection identified non-microsatellite repeats in various CRMs. Combinations of recurring motifs and/or evolutionarily conserved TF binding sites have guided the identification of CRMs active in the *Ciona* muscle [21,39–42] and central nervous system (CNS) [41,43], as well as in various tissues/embryonic territories of *Drosophila* [10,44,45] and in the zebrafish notochord [46]. For these reasons, we sought to investigate whether these repeats could aid in the prediction of novel notochord CRMs in *Ciona intestinalis*. We noticed that Ci-CRM90 features two nearly identical 73-bp sequence blocks, each containing two copies of a smaller 20-bp repeat; moreover, a sequence motif related to the 20-bp repeat was found in Ci-CRM9 (S4 Fig). Ci-CRM26 contains a 19-bp tandem repeat, whose first copy overlaps with the E-box required for activity. The exact sequences of both of these repeats are unique in the *Ciona intestinalis* genome; however, shorter variations of the Ci-CRM26 repeat are seen in four other notochord CRMs (S4 Fig). To assess the predictive ability of functional binding sites and motifs, we tested 36 genomic fragments containing arrangements of binding sites and/or motifs identical or similar to those found in the Ci-CRMs (Fig 1). We only detected notochord expression in one construct (S3 Table, S4 Table): the short motif found in Ci-CRM26, which occurs ~3,017 times in the *Ciona intestinalis* genome, led us to the identification of a novel notochord CRM within the *Ci-Noto2* locus (S4 Fig and S4 Table).

We also tested whether interspecific sequence homology could improve the prediction of notochord CRMs, since evolutionary conservation is widely used to pinpoint *Ciona cis*-regulatory regions (*e*.*g*., [47–49]). The CRMs presented here were isolated using a conservation-independent approach, but when we retrospectively assessed this parameter, we observed surprising interspecific variability among their sequences. Indeed, many of these *Ciona intestinalis* CRMs display limited conservation, if any, with *Ciona savignyi* (S4 Fig). In addition, even though some binding sites, such as the Ci-Fox and E-box sites of Ci-CRM76, are perfectly conserved between the two *Ciona* species, neither is required for activity (S4 Fig); this suggests that even interspecifically conserved notochord TF binding sites are not reliable indicators of functionality. These results concur with studies in *Drosophila* that suggest that clustered binding sites within CRMs might be retained over evolution for reasons other than selection or functional necessity [12].

**Fig 4:**
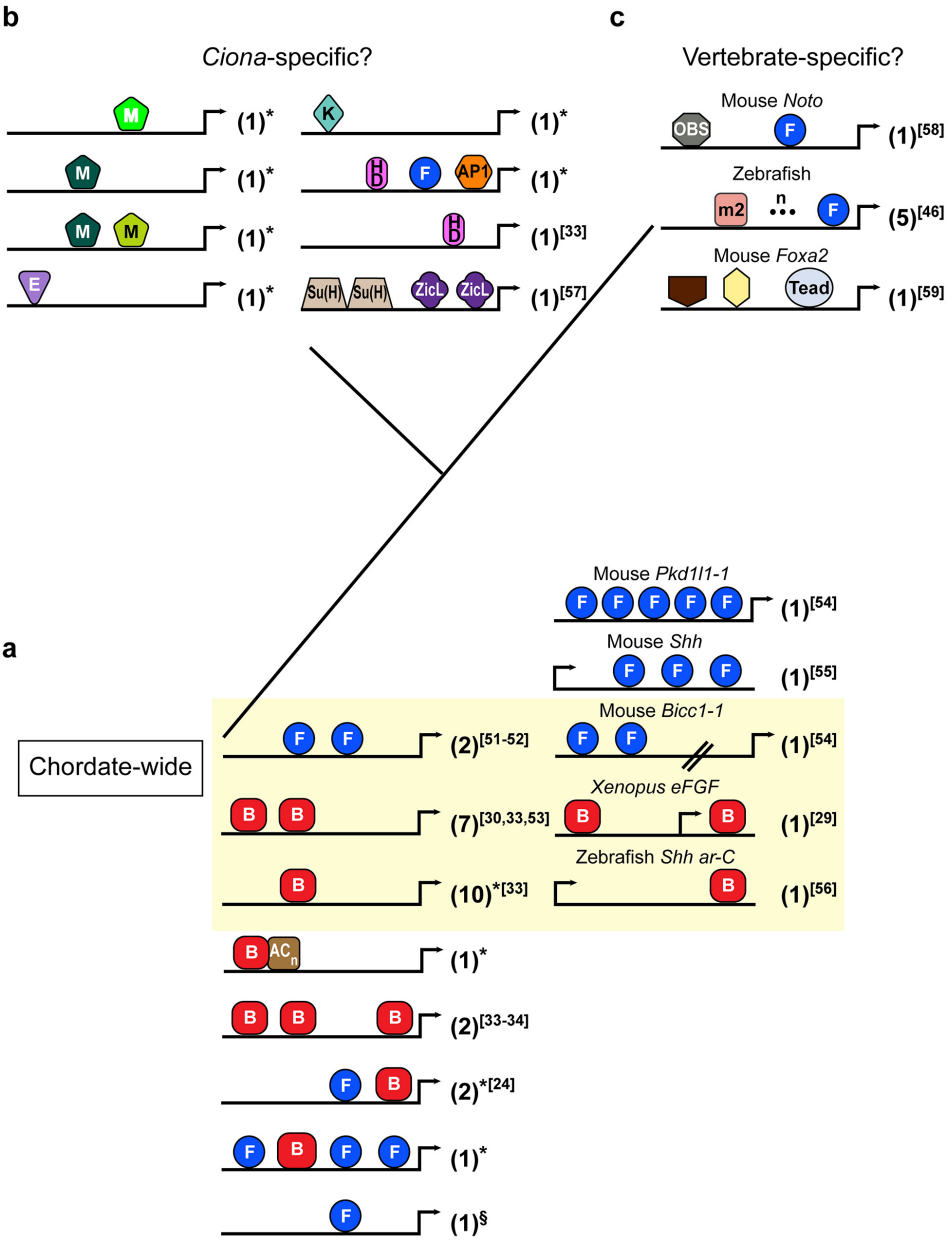
The *cis*-regulatory building blocks of notochord CRMs in chordate phylogeny. Schematic representation of all 46 experimentally validated and fully characterized notochord CRMs published in any chordate [24,29,30,33,34,46,51–59], grouped into 24 structural types. Among the 35 *Ciona* CRMs, 14 were described for the first time in this study (Fig 1). Notochord CRMs are symbolized by black lines, with arrows representing transcription start sites. Colored shapes depict putative transcription factor binding sites. Only experimentally validated binding sites required for the *in vivo* activity of each CRM are reported. The numbers in parentheses denote the number of related CRMs identified thus far that display each *cis*-regulatory arrangement. **a:** Chordate-wide *cis*-regulatory features of *Ciona* notochord CRMs (left column) and vertebrates (right column). The area highlighted in yellow encompasses notochord CRMs from *Ciona* and from vertebrates that show directly comparable binding sites and arrangements. Notochord CRMs above and below the yellow area rely on either reiterative or alternate configurations of Brachyury (B) and Foxa2 (F) functional binding sites. **b:** Notochord CRMs that, thus far, do not seem to have counterparts in other chordates, and are therefore tentatively classified as *Ciona*-specific. **c:** Notochord CRMs that currently do not appear to have counterparts in *Ciona* or other invertebrate chordates and are provisionally classified as vertebrate-specific. TF binding sites are abbreviated as follows: AP1: activator protein 1, B: Brachyury, F: Fox, E: E-box, HD: homeodomain, K: Krüppel-like, M, Myb-like, m2, Motif 2, OBS: orphan binding site. A brown pentagon and a yellow hexagon in the mouse *Foxa2* notochord CRM indicate required orphan binding sites. * this study; § notochord CRM associated with the *Ci-quaking* gene (KH.S115.4) [60].

In sum, the unexpected variety and flexibility of the mechanisms that we have described here limited our ability to predict notochord CRMs from sequence alone. Yet, although our results seem to question the existence of a straightforward notochord *cis*-regulatory code, this study uncovered recurring grammatical elements shared by notochord CRMs. In particular, Brachyury and Foxa2 binding sites emerge as the basic building blocks of most *Ciona* notochord CRMs (Fig 4A), and these results are consistent with findings in other chordates. In fact, Brachyury binding sites have been found to be critical for the function of notochord in different animals (*e*.*g*. [29,50]), and our previous studies in *Ciona* show that they can act either individually or cooperatively [33,34,53]. Their association with (AC) microsatellites in Ci-CRM66 and in the mouse genome [26] might represent a recurring feature of a distinct class of notochord CRMs (Fig 4A). Foxa2 sites are required in notochord CRMs from zebrafish and mice [46,54], although they are rarely sufficient to initiate expression when in single copy, and often necessitate additional sequences [46,58,61] whose identity appears to be lineage-specific (Fig 4A and 4C). These observations and our previous results [33] reflect the reported pioneer chromatin-opening ability of Fox proteins [62], which may not able to activate gene expression *per se* but are required to increase the accessibility of CRMs to other transcription factors, such as Brachyury and/or other notochord-specific activators.

The basic *cis*-regulatory repertoire that we have uncovered was likely expanded *via* vertebrate-specific evolutionary events; such events include the notochord deployment of additional TFs, such as homeobox and Hox proteins and their co-factors, which are remarkably underrepresented in the tunicate notochord, [63] along with the duplication and consequent divergence of regulatory regions.

## Materials and Methods

### Embryo culture, fixation, electroporation and staining

Adult *Ciona intestinalis* were purchased from Marine Research and Educational Products (M-REP; Carlsbad, CA) and kept in an aquarium in recirculating artificial sea water at 17–18°C. Culturing and electroporations were carried out as previously described [64]. After electroporation, transgenic embryos were fixed in 0.2% glutaraldehyde and stained at 37°C with 5-bromo-4-chloro-3-indolyl-β-D-galactopyranoside (X-gal) [64]. Stained embryos were washed in 500 μL PBST (1X PBS, 0.1% Tween 20), post-fixed in 300–500 μL of 4% paraformaldehyde in PBST, and stored at 4°C. To determine the comparative activities of wild-type and mutated constructs, the proportions of X-gal stained embryos exhibiting notochord staining were determined from at least three independent experiments. Data presented in graphs represent average values, with error bars denoting the standard deviation.

### Plasmid construction

Genomic fragments for enhancer discovery and analyses were cloned into the pFBΔSP6 plasmid, which contains the *LacZ* reporter gene [64]. After the initial characterization of each notochord CRM, subsequent deletions and mutations were made either by utilizing unique restriction enzyme sites or by Polymerase Chain Reaction (PCR), using the smallest active DNA fragment as a template. A list of the oligonucleotides employed for PCR amplifications and the restriction sites used for cloning the most relevant constructs is provided in S5 Table.

For the predictions of notochord CRMs, suitable genomic regions were first identified by searching either the *Ciona* genome or a database of validated *Ciona* notochord genes for transcription factor binding sites, motifs or other sequence signatures present in notochord CRMs, using the GUFEE program [24]. Our database of *Ciona* notochord genes contained the sequences of the putative genomic loci of 300 notochord genes. We manually annotated the gene models from expression data present in the ANISEED database [38] and from our results. The sequences included in the database were extracted from the UCSC genome browser (*Ciona intestinalis* version 1) by Dr. John R. Edwards (Washington University, St. Louis).

## Supporting Information

S1 Fig Initial characterization of a subset of the notochord CRMs described in Fig 1. **a-f:** Schematic representations of wild-type notochord CRMs and site-specific mutants of selected binding sites (see Fig 1 for key). Maroon bars represent constructs capable of directing notochord expression of the *LacZ* reporter, while inactive configurations are depicted by gray bars. Mutagenized sites are colored in white and marked by “X” signs, and the mutant sequences are shown in red. Each panel contains microphotographs of representative transgenic embryos carrying selected plasmids. Colored arrowheads indicate stained domains as follows: red: notochord, blue: CNS, yellow: endoderm, orange: muscle, purple: mesenchyme, green: epidermis. Graphs display the percentage of embryos showing notochord staining among all stained embryos; error bars indicate the standard deviation. Abbreviations: B, Brachyury; E, E-box (presumptive bHLH binding site); F, Fox; HD, homeodomain; M, Myb-like; S/K1, Sp1/Klf.
(TIF)

S2 Fig Deletion/mutation analysis of the notochord CRMs described in Fig 2. **a,b,d:** Schematic representations of wild-type notochord CRMs and site-directed mutants of the binding sites shown in the key (top right). The color-coding of the bars representing the DNA regions is the same as in Fig 2. “X” signs indicate mutagenized sites, and mutant sequences are shown in red. **c,e,f:** Representative embryos carrying a selection of the plasmids depicted in **a,b,d**. Colored arrowheads indicate stained domains as in S1 Fig. Abbreviations: AP1: Activator protein 1, Bra: Brachyury, HD: homeodomain.
(TIF)

S3 Fig Deletion/mutation analysis of the notochord CRMs described in Fig 3 and of Ci-CRM9. **a-e:** (Left) Schematic representations of notochord CRMs and their mutant versions. Putative binding sites are depicted as shown in the key on the top right in **a**. Color-coding is as in S1 Fig. Mutagenized sites are indicated by “X” signs and the mutant sequences are in red. (Middle) Transgenic embryos carrying a selection of informative plasmids. Arrowheads are color-coded as in S1 Fig. Note that in **c** the B4 mutation was inserted in the 749-bp fragment since the minimal CRM (547-bp) exhibits a less consistent staining pattern. (Right, **b-e)** Quantification of notochord stained embryos harboring either wild-type or mutant constructs; error bars denote the SD. Abbreviations are as in S1 Fig.
(TIF)

S4 Fig Architectural constraints, sequence motifs and interspecies conservation of *Ciona* notochord CRMs. Related to Figs 1 and 3. **a-d:** Impact of the alteration of structural features on the function of Ci-CRM76 in notochord cells. (Left) schematic representations of wild-type (WT) and mutant versions of Ci-CRM76 containing the changes in enhancer architecture highlighted in red. Putative Myb-like binding sites are named as in S1 Fig. Symbols for all other binding sites are as in Fig 1. The necessary Myb-like sites are marked by “1” and “2”. Arrows show the orientation of the binding sites of interest. (Right) Representative transgenic embryos obtained from the same batch of animals, harboring the plasmids summarized at their left. Arrowheads mark stained territories, as in S1 Fig. The percentage of embryos exhibiting notochord staining is reported in the lower right corner. M: Myb-like binding site. **e-g:** Sequence motifs shared by subsets of notochord CRMs. **e,f:** Schematic representations of notochord CRMs sharing distinctive sequence blocks. Tan bars symbolize notochord CRMs and diagonal parallel lines depict genomic regions that are present in the constructs but omitted from the figure for clarity. In Ci-CRM90, a 73-bp sequence, boxed in yellow, is imperfectly repeated in the 245-bp region shown here. Within this 73-bp sequence, four motifs were identified (#1–4) using the MEME software (http://meme.nbcr.net). A related motif was identified in the Ci-CRM9 sequence (boxed in yellow), adjacent to the Ci-Bra binding site necessary for its function. The sequences of all these motifs, and the derived consensus, are reported on the right. **f:** Another motif (light blue boxes) was found to be present in one or two copies in a different subset of CRMs. The sequences of its iterations, and the derived consensus, are reported on the right. The distances between the necessary site(s) and each motif are shown, unless they overlap. A closely related motif was found in Ci-CRM99. The CRMs included in this figure are depicted in a slightly different scale compared to the previous figures, to provide a more accurate representation of the distances among binding sites. **g:** Microphotograph of a transgenic embryo electroporated with the *Ci-Noto2* notochord CRM, which was predicted using the Ci-CRM26 motif. **h,i:** Variability in the interspecific conservation of notochord CRMs sequences between *Ciona intestinalis* and *Ciona savignyi*. (Top) VISTA plots (http://pipeline.lbl.gov/cgi-bin/gateway2) illustrating the sequence conservation across the “full-length” *Ci-Fkbp9* (**h**) and Ci-CRM76 (**i**) notochord CRMs between *Ciona intestinalis* (*Ci*) and *Ciona savignyi* (*Cs*), obtained utilizing the following parameters: calculation window, 80 bp; minimum conservation width, 50 bp; conservation identity, 70%. Conserved non-coding regions are depicted as pink peaks, conserved coding regions as blue peaks. The areas corresponding to the minimal CRMs identified and described in Fig 1 are boxed in red. (Bottom) Sequence alignment of the *Ci* minimal notochord CRMs with the corresponding regions of *Cs*. In *Ci*, binding sites are highlighted as in Fig 1, whereas related non-syntenic putative binding sites, whenever present, are indicated in lighter colors in the *Cs* sequence.
(TIF)

S1 Table Genomic locations of minimal notochord CRMs. (DOCX)

S2 Table Properties of (AC) microsatellite clusters tested for notochord activity. (DOCX)

S3 Table Properties of genomic regions near notochord genes showing arrangements of sites resembling those found in selected notochord CRMs. (DOCX)

S4 Table Properties of genomic regions containing sequence motifs found in subsets of notochord CRMs. (DOCX)

S5 Table Primers utilized for the PCR amplification of the most relevant constructs used for CRM characterization. (DOCX)
