# Supplementary figures and images for: Brachyury, Foxa2 and the cis-Regulatory Origins of the Notochord
Source: PLoS Genet. 2015 Dec 18;11(12):e1005730. doi: 10.1371/journal.pgen.1005730 (PMC4684326; doi:10.1371/journal.pgen.1005730)

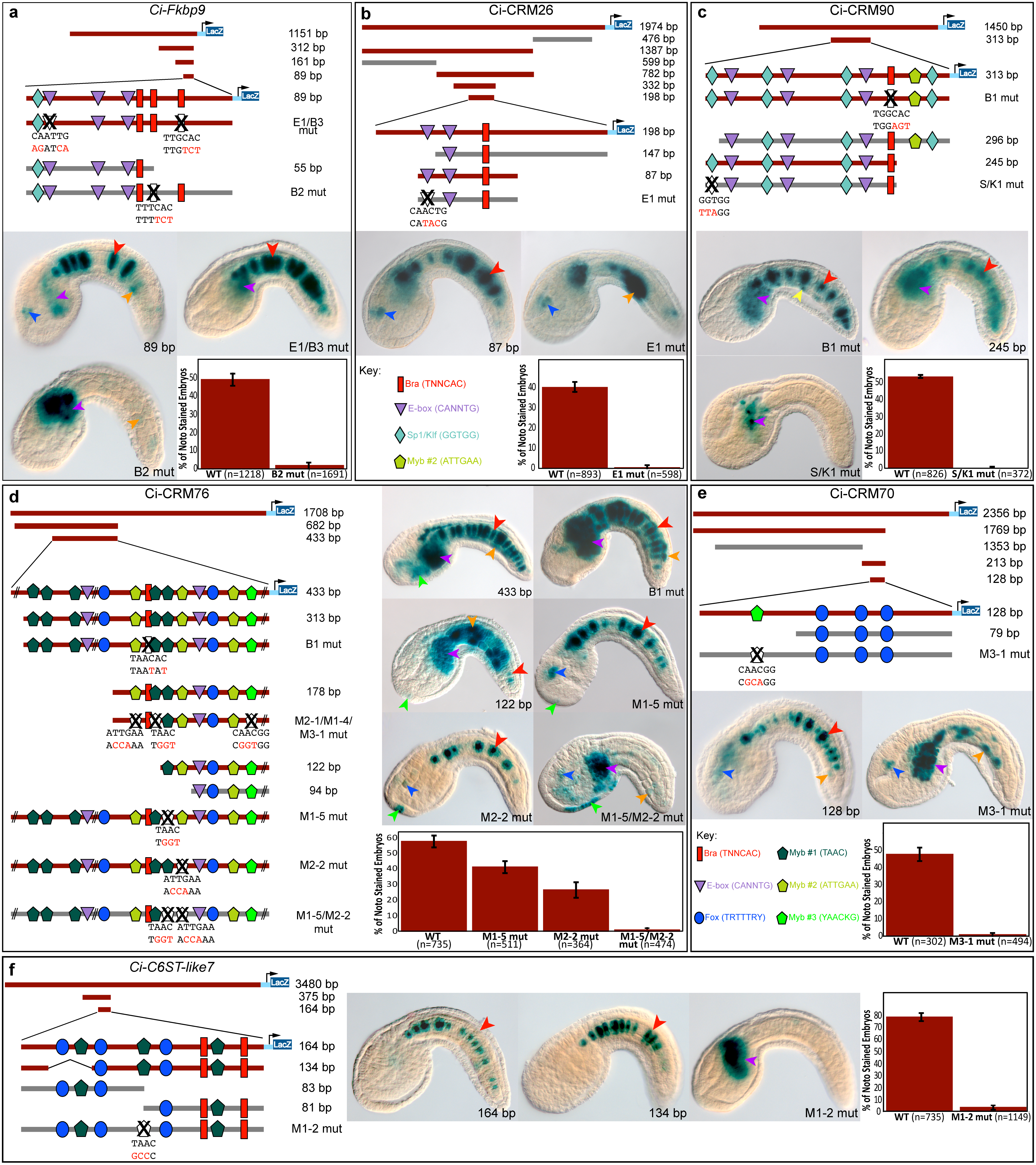

Supplement: S1 Fig — a-f: Schematic representations of wild-type notochord CRMs and site-specific mutants of selected binding sites (see Fig 1 for key). Maroon bars represent constructs capable of directing notochord expression of the LacZ reporter, while inactive configurations are depicted by gray bars. Mutagenized sites are colored in white and marked by “X” signs, and the mutant sequences are shown in red. Each panel contains microphotographs of representative transgenic embryos carrying selected plasmids. Colored arrowheads indicate stained domains as follows: red: notochord, blue: CNS, yellow: endoderm, orange: muscle, purple: mesenchyme, green: epidermis. Graphs display the percentage of embryos showing notochord staining among all stained embryos; error bars indicate the standard deviation. Abbreviations: B, Brachyury; E, E-box (presumptive bHLH binding site); F, Fox; HD, homeodomain; M, Myb-like; S/K1, Sp1/Klf. (TIF) [file pgen.1005730.s001.tif]

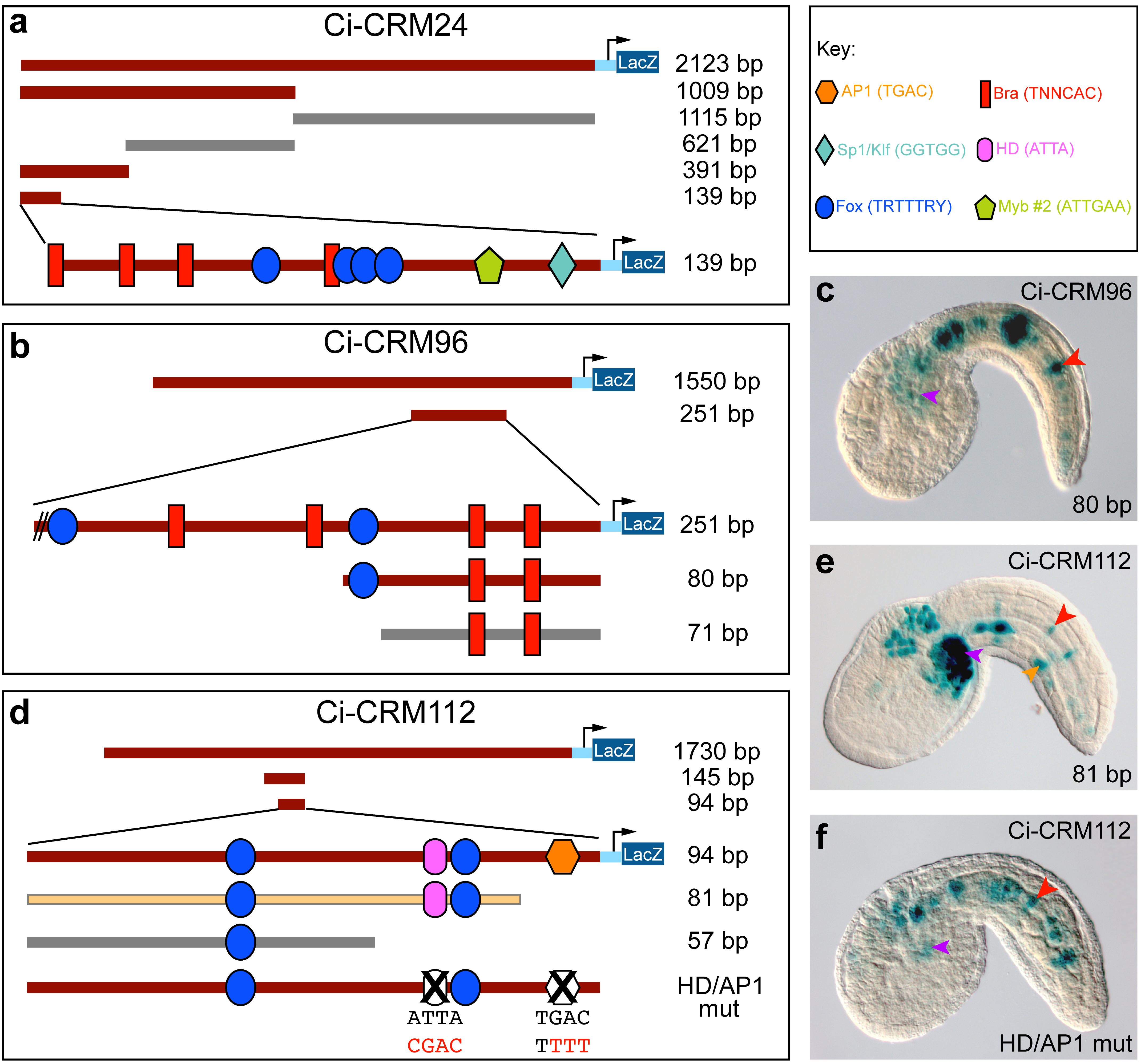

Supplement: S2 Fig — a,b,d: Schematic representations of wild-type notochord CRMs and site-directed mutants of the binding sites shown in the key (top right). The color-coding of the bars representing the DNA regions is the same as in Fig 2. “X” signs indicate mutagenized sites, and mutant sequences are shown in red. c,e,f: Representative embryos carrying a selection of the plasmids depicted in a,b,d. Colored arrowheads indicate stained domains as in S1 Fig. Abbreviations: AP1: Activator protein 1, Bra: Brachyury, HD: homeodomain. (TIF) [file pgen.1005730.s002.tif]

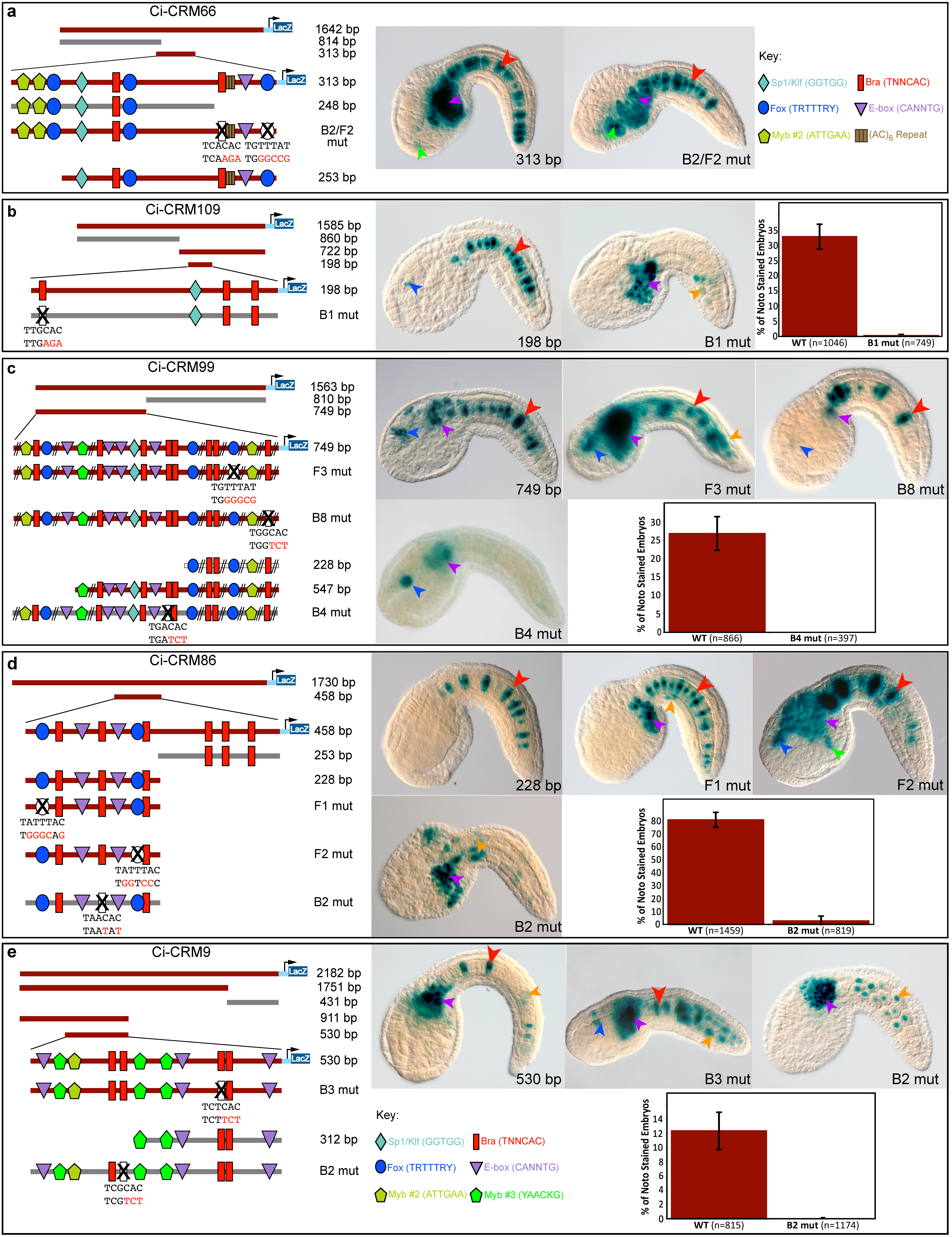

Supplement: S3 Fig — a-e: (Left) Schematic representations of notochord CRMs and their mutant versions. Putative binding sites are depicted as shown in the key on the top right in a. Color-coding is as in S1 Fig. Mutagenized sites are indicated by “X” signs and the mutant sequences are in red. (Middle) Transgenic embryos carrying a selection of informative plasmids. Arrowheads are color-coded as in S1 Fig. Note that in c the B4 mutation was inserted in the 749-bp fragment since the minimal CRM (547-bp) exhibits a less consistent staining pattern. (Right, b-e) Quantification of notochord stained embryos harboring either wild-type or mutant constructs; error bars denote the SD. Abbreviations are as in S1 Fig. (TIF) [file pgen.1005730.s003.tif]

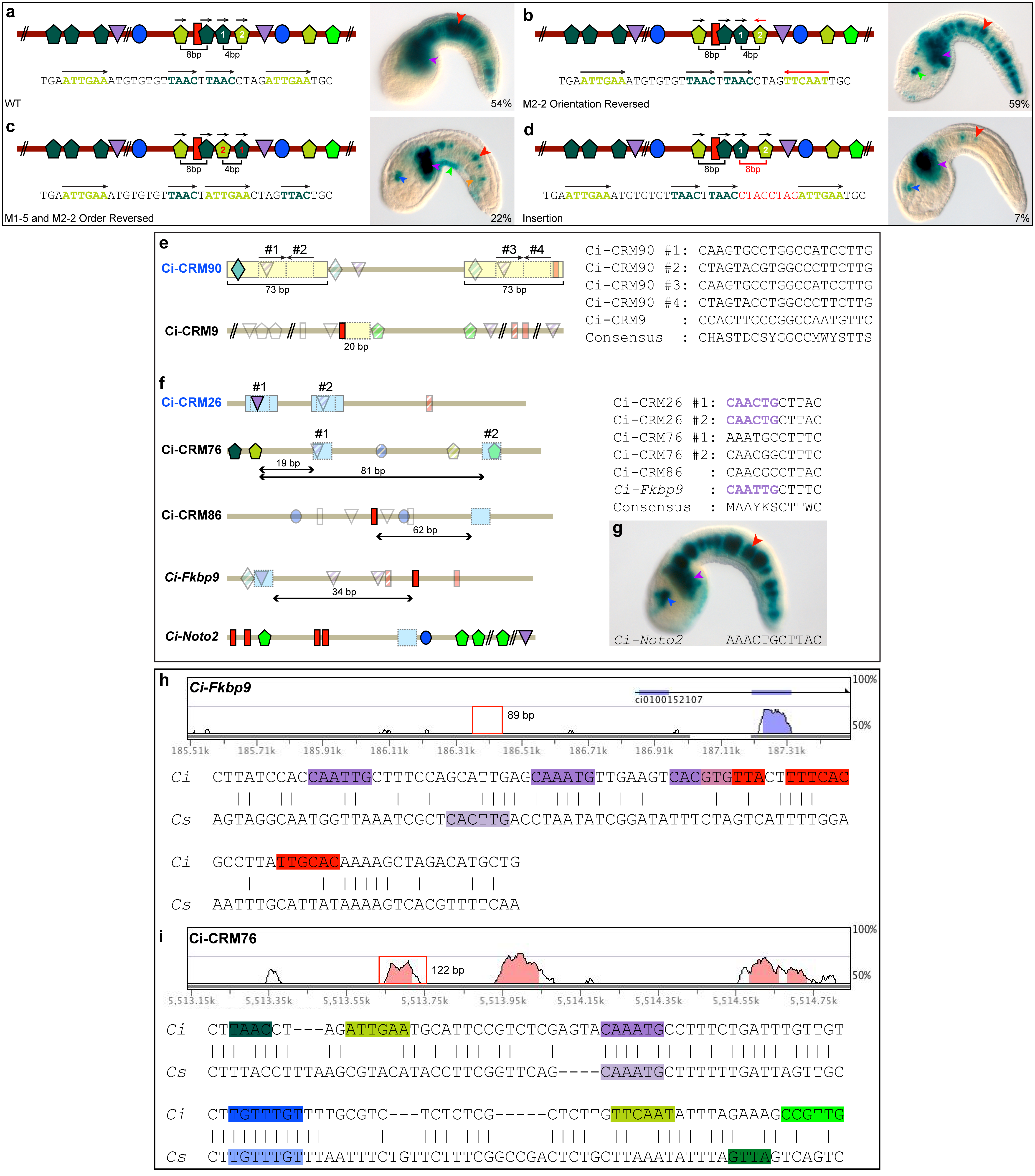

Supplement: S4 Fig — Related to Figs 1 and 3. a-d: Impact of the alteration of structural features on the function of Ci-CRM76 in notochord cells. (Left) schematic representations of wild-type (WT) and mutant versions of Ci-CRM76 containing the changes in enhancer architecture highlighted in red. Putative Myb-like binding sites are named as in S1 Fig. Symbols for all other binding sites are as in Fig 1. The necessary Myb-like sites are marked by “1” and “2”. Arrows show the orientation of the binding sites of interest. (Right) Representative transgenic embryos obtained from the same batch of animals, harboring the plasmids summarized at their left. Arrowheads mark stained territories, as in S1 Fig. The percentage of embryos exhibiting notochord staining is reported in the lower right corner. M: Myb-like binding site. e-g: Sequence motifs shared by subsets of notochord CRMs. e,f: Schematic representations of notochord CRMs sharing distinctive sequence blocks. Tan bars symbolize notochord CRMs and diagonal parallel lines depict genomic regions that are present in the constructs but omitted from the figure for clarity. In Ci-CRM90, a 73-bp sequence, boxed in yellow, is imperfectly repeated in the 245-bp region shown here. Within this 73-bp sequence, four motifs were identified (#1–4) using the MEME software (http://meme.nbcr.net). A related motif was identified in the Ci-CRM9 sequence (boxed in yellow), adjacent to the Ci-Bra binding site necessary for its function. The sequences of all these motifs, and the derived consensus, are reported on the right. f: Another motif (light blue boxes) was found to be present in one or two copies in a different subset of CRMs. The sequences of its iterations, and the derived consensus, are reported on the right. The distances between the necessary site(s) and each motif are shown, unless they overlap. A closely related motif was found in Ci-CRM99. The CRMs included in this figure are depicted in a slightly different scale compared to the previous figure [file pgen.1005730.s004.tif]
