# Supplementary material for: Brachyury, Foxa2 and the cis-Regulatory Origins of the Notochord
Source: PLoS Genet. 2015 Dec 18;11(12):e1005730. doi: 10.1371/journal.pgen.1005730 (PMC4684326; doi:10.1371/journal.pgen.1005730)
Supplement: S1 Table — (DOCX) [file pgen.1005730.s005.docx]

| **Table S1: Genomic locations of minimal notochord CRMs** | | | | | |
| --- | --- | --- | --- | --- | --- |
| **CRM** | **Size (bp)** | **Genomic Coordinates** | **Nearest Gene**  **Model** | **CRM Location Relative to Gene Model** | **Notochord Expression of Closest Gene** |
| Ci-CRM96 | 80 | KhL5:133,335-133,414 | KH.L5.8  *Pavarotti-like* | intronic | Yes^a^ |
| Ci-CRM24 | 139 | KhC9:5,648,367-5,648,505 | KH.C9.371  *Ddr1,Ddr2, Musk* | upstream | Yes^b^ |
| Ci-CRM112 | 94 | KhC7:40,900-40,993 | KH.C7.500  *Noe2,Olm2A/B* | upstream | Yes^a^ |
| Ci-CRM66 | 253 | KhC7:3,853,970-3,854,222 | KH.C7.568  *Ephrin3* | intronic | Yes^c^ |
| Ci-CRM9 | 530 | KhL13:47,071-47,600 | KH.L13.2 | upstream | Yes^a^ |
| Ci-CRM86 | 228 | KhL65:311,070-311,303 | KH.L65.10  *Dryk2/3/4* | intronic | Yes^d^ |
| Ci-CRM99 | 547 | KhL128:214,483-215,027 | KH.L128.2  *Furin,Nec1/2* | intronic | No |
| Ci-CRM109 | 198 | KhS610:12,703-12,901 | KH.S610.5 *Kelch-rel*. | downstream | No |
| *Ci-Fkbp9* | 89 | KhC9:35,651-35,739 | KH.C9.778  *Ci-Fkbp9* | upstream | Yes^d^ |
| Ci-CRM26 | 87 | KhC10:2,816,564-2,816,650 | KH.C10.320  *Ubp4,Ubp8,Ubp11* | downstream | No |
| Ci-CRM90 | 245 | KhS345:68,522-68,718 | KH.S345.4 | upstream | No |
| Ci-CRM76 | 122 | KhC5:4,472,021-4,472,163 | KH.C5.399  *Rfx1/2/3* | downstream | Yes^e^ |
| Ci-CRM70 | 128 | KhC12:712,425-712,552 | KH.C12.16  *CbpD,CbpE,CbpN* | downstream | Yes^e^ |
| *Ci-C6ST-like7* | 164 | KhC1:9,941,324-9,941,487 | KH.C1.738  *Ci-C6ST-like7* | upstream/  5′ UTR | Yes^f^ |
| ^a^ Our unpublished results.  ^b^ [1] ^c^ [2] ^d^ [3] ^e^ [4] ^f^ [5] | | | | | |

Abbreviations: bp: base pairs, rel.: related.
