# Supplementary material for: Brachyury, Foxa2 and the cis-Regulatory Origins of the Notochord
Source: PLoS Genet. 2015 Dec 18;11(12):e1005730. doi: 10.1371/journal.pgen.1005730 (PMC4684326; doi:10.1371/journal.pgen.1005730)
Supplement: S2 Table — (DOCX) [file pgen.1005730.s006.docx]

| **Table S2. Properties of (AC) microsatellite clusters tested for notochord activity** | | | | | | |
| --- | --- | --- | --- | --- | --- | --- |
| **Genomic Coordinates** | **Size**  **(bp)** | **# of repeats** | **Adjacent Ci-Bra site** | **Ci-Bra occ.^a^** | **Nearby noto.**  **gene** | **Cluster noto. activity** |
| KhC7:3,853,970-3,854,222  (Ci-CRM66) | 253 | 6 | TCACAC | (+++) | Yes^b^  KH.C7.568  *Ephrin3* | Yes |
| KhC1:7,533,575-7,533,808 | 233 | 13 | TCACAC | (+) | No | No |
| KhC3:1,909,915-1,910,349 | 434 | 3^c^ | TCACAC | (++) | No | No |
| KhC4:4,454,695-4,454,984 | 289 | 6 | TCACAC | (++) | No | No |
| KhC5:2,406,810-2,407,177 | 367 | ≥6 | TTGCAC | (+++) | No | No |
| KhC5:3,514,633-3,515,030 | 397 | 7 | TCACAC | (+) | No | No |
| KhC9:1,537,164-1,537,608 | 444 | 7 | TCACAC | (++) | No | No |
| KhC12:3,856,661-3,857,122 | 461 | 17 | TCACAC | (++) | No | No |
| KhL24:269,249-269,597 | 348 | 6 | TCACAC | (++) | No | No |
| KhC8:5,801,833-5,802,128* | 295 | 6 | TTACAC | (+) | Yes^d^  KH.C8.210 | No |
| KhC5:4,916,567-4,916,815 | 248 | 33 | N/A | (++) | Yes^d,e^  KH.C5.217  *Ci-Noto14* | No |
| KhC2:3,233,971-3,234,248 | 277 | 9 | N/A | (++) | Yes^f^  KH.C2.327  *Ci-AFF* | No |
| ^a^ Data compiled from [6]; 0<(+)<1; 1<(++)<2; (+++)>2 fold enrichment based upon the highest peaks within 250 bp of each construct. The number of (AC) repeats in each region varies in different versions of the genome.  ^b^ [2]  ^c^ While six repeats were predicted from the genomic information, a polymorphism in the actual amplified sequence reduced the repeat number to three.  ^d^ [4]  ^e^ [7]  ^f^ [8]  *The (AC) repeat is masked in the current Aniseed genome browser, but is visible in the current UCSC genome browser using coordinates chr08q:6,421,219-6,421,514  Abbreviations: bp: base pairs, Bra.: Brachyury, occ.: occupancy, noto.: notochord. | | | | | | |
