# Supplementary material for: Brachyury, Foxa2 and the cis-Regulatory Origins of the Notochord
Source: PLoS Genet. 2015 Dec 18;11(12):e1005730. doi: 10.1371/journal.pgen.1005730 (PMC4684326; doi:10.1371/journal.pgen.1005730)
Supplement: S3 Table — (DOCX) [file pgen.1005730.s007.docx]

| **Table S3. Properties of genomic regions near notochord genes showing arrangements of sites resembling those found in selected notochord CRMs** | | | | | | | |
| --- | --- | --- | --- | --- | --- | --- | --- |
| **CRM** | **Genomic Coordinates** | **Size**  **(bp)** | **Ci-Bra occ. ^a^** | **Ci-FoxA-a occ. ^a^** | **Differences from CRM^b^** | **Nearby noto. gene** | **Noto. activ.** |
| Ci-CRM76 | KhL130:13,791-13,989 | 198 | (++) | (+) | None | KH.L130.1^c^  *ITA3,ITA6,ITA7* | No |
|  | KhL22:339,714-339,950 | 236 | (+) | (++) | None | KH.L22.45^d^  *EGFR-a* | No |
|  | KhC7:3,237,042-3,237,319 | 277 | (+) | (+) | None | KH.C7.128^e^  *ABL1,FAK1, FAK2* | No |
|  | KhC9:316,897-317,075 | 178 | (-) | (+) | None | KH.C9.118^e^ | No |
|  | KhC9:928,192-928,333 | 141 | (++) | (-) | None | KH.C9.72^f^  *Cadherin8* | No |
|  | KhC4:2,310,149-2,310,299 | 150 | (+++) | (+) | None | KH.C4.391^g^  *FRMD1/6,Ptn13* | No |
|  | KhC1:692,941-693,124 | 183 | (+) | (-) | None | KH.C1.1039^e^  *HGFA,NETR,Tmps3* | No |
|  | KhC3:6,326,824-6,327,017 | 193 | (+) | (+) | Sp., Ord. | KH.C3.21^h^  *Ci-perlecan* | No |
| Ci-CRM96 | KhC2:1,654,484-1,654,661 | 177 | (+++) | (+) | None | KH.C2.725^g^  *Su(H)/RBP-J* | No |
|  | KhS534:18,581-18,729 | 148 | (++) | (+) | SS (F) | KH.S534.2^e^  *Rbcc1* | No |
|  | KhS862:20,795-20,996 | 201 | (++) | (+) | SS (F) | KH.S862.1^i^  *Camk1* | No |
|  | KhC3:4,160,230-4,160,464 | 234 | (+) | (++) | SS (F) | KH.C3.40^e^  *WDR90* | No |
|  | KhC1:848,738-848,977 | 239 | (+) | (+) | SS (F), Ord. | KH.C1.10^e^  *LIM & SH3 protein* | No |
| Ci-CRM112^j^ | KhC12:4,849,888-4,850,067 | 179 | (+) | (++) | Ori. (AP1) | KH.C12.129^k^  *Ci-ERM* | No |
|  | KhC1:1,742,955-1,743,191 | 236 | (+) | (++) | Ori. (HD),  SS (F) | KH.C1.832^k^  *Ci-fibrn* | No |
|  | KhC13:1,720,410-1,720,574 | 164 | (+) | (++) | Ori. (HD),  SS (F) | KH.C13.73^l^  *Rapostlin* | No |
|  | KhC3:4,417,287-4,417,495 | 208 | (+) | (+) | Sp. | KH.C3.487^g^  *Mot12,Mot2* | No |
|  | KhC5:385,641-385,824 | 183 | (+) | (+) | Sp. | KH.C5.128^g^  *PDE4A,PDE4B,PDE4D* | No |
|  | KhC14:1,306,308-1,306,481 | 173 | (+) | (++) | Sp. | KH.C14.88^m^  *Cadherin-related-6* | No |
|  | KhC3:6,401,942-6,402,151 | 209 | (+) | (+) | Sp. | KH.C3.225^e^  *Fbn1/2/3,Ltbp4* | No |
|  | KhC7:4,905,980-4,906,113 | 133 | (+) | (++) | Ord., Sp.  Ori. (HD, F, AP1) | KH.C7.781^g^  *Fgd1/2/4* | No |
|  | KhC12:4,670,577-4,670,725 | 148 | (++) | (++) | Sp., Ord. | KH.C12.487^g^  *Ctrb1,HGFA,NETR,Tmps2* | No |
| Ci-CRM24^j^ | KhL119:179,207-179,383 | 176 | (++) | (+) | Ori. (F1),  SS (F1, B4, F2) | KH.L119.9^e^  *Ptcd1* | No |
|  | KhC11:1,520,981-1,521,144 | 164 | (+) | (++) | Ori. (F2, F3), Sp., SS (F1-F3, B4) | KH.C11.456^e,h^  *Myh9/10/11* | No |
|  | KhC13:1,609,016-1,609,294 | 278 | (+) | (++) | Sp.,  SS (F1, F2, F3) | KH.C13.116^h^  *Ci-Collagen alpha 1-2* | No |
|  | KhC9:36,593-36,720 | 127 | (+) | (+) | Ord., Sp.,  SS (F1, F2, F3) | KH.C9.778^e^  *Ci-Fkbp9* | No |
|  | KhC9:36,593-36,919 | 326 | (+) | (+) | Sp.,  SS (F1- F3) | KH.C9.778^e^  *Ci-Fkbp9* | No |
|  | KhS417:28,334-28,769 | 435 | (+) | (++) | Sp., Ori. (F1), SS (F1,F3) | KH.S417.6^f^  *Ci-fibronectin* | No |
| ^a^Data compiled from [6]; (-), no apparent signal; 0<(+)<1; 1<(++)<2; (+++)>2 fold enrichment based upon the highest peaks within 250 bp of each construct.  ^b^Our database of genomic regions surrounding notochord genes was searched for areas containing the nucleotides, spacing and orientation of the putative transcription factor binding sites necessary for the notochord activity of the example CRM. Architectural changes from the query CRM are listed with the differing site(s) denoted in parentheses where appropriate.  ^c^[3] ^d^[2] ^e^[4] ^f^[9] ^g^Our unpublished results. ^h^[7] ^i^[10] ^j^No exact matches for this CRM within notochord gene database. ^k^[11] ^l^[1] ^m^[12] ^n^[13]  Abbreviations: bp: base pairs, occ.: occupancy, noto.: notochord, clust: cluster, act.: activity, Sp.: spacing, Ord.: order, SS: site sequence, Ori.: orientation, F: Fox, HD: Homeodomain, B/Bra.: Brachyury. | | | | | | | |
