# Supplementary material for: Brachyury, Foxa2 and the cis-Regulatory Origins of the Notochord
Source: PLoS Genet. 2015 Dec 18;11(12):e1005730. doi: 10.1371/journal.pgen.1005730 (PMC4684326; doi:10.1371/journal.pgen.1005730)
Supplement: S4 Table — (DOCX) [file pgen.1005730.s008.docx]

| **Table S4. Properties of genomic regions containing sequence motifs found in subsets of notochord CRMs** | | | | | | | |
| --- | --- | --- | --- | --- | --- | --- | --- |
| **Motif** | **Construct Genomic Coordinates** | **Size**  **(bp)** | **Ci-Bra occ. ^a^** | **Ci-FoxA-a occ. ^a^** | **Nearby noto. gene** | **Clust. noto. activity** |  |
| CHASTDCSYGG-CCMWYSTTS^b^ | KhC14:1,054,606-1,055,224 | 618 | (+) | (+) | No | No |  |
| MAAYKSCTTWC^b^ | KhL97:14,732-15,037 | 305 | (+) | (+) | Yes^c^  KH.L97.4  *PURA1/2* | No |  |
|  | KhC14:3,858,124-3,858,879 | 755 | (+) | (++) | Yes^d^  KH.C14.116  *Matn2* | No |  |
|  | KhC1:5,920,987-5,921,271 | 284 | (++) | (++) | Yes^d,e^  KH.C1.330  *Ci-Noto15* | No |  |
|  | KhC2:263,513-264,132 | 619 | (+) | (-) | Yes^f^  KH.C2.420  *Ci-PTP* | No |  |
|  | KhS115:117,337-118,152 | 815 | (++) | (+) | Yes^g^  KH.S115.4  *Ci-Quaking* | No |  |
|  | KhC11:4,181,166-4,181,690 | 525 | (+++) | (++) | Yes^f^  KH.C11.328  *Ci-Noto2* | Yes^b^ |  |
| MAAYKSCTTWC and Bra(TT core)  (*Ci-Fkbp9*-like)^h^ | KhS698:15,840-16,399 | 559 | (+) | (+) | No | No |  |
| ^a^Data compiled from [6]; (-), no apparent signal; 0<(+)<1; 1<(++)<2; (+++)>2-fold enrichment based upon the highest peaks within 250 bp of each construct  ^b^See Figure S4 for more details.  ^c^[4] ^d^[3] ^e^[14] ^f^[11] ^g^[9]  ^h^The genome was searched for areas containing the same order, spacing and orientation as the MAAYKSCTTWC motif and TTTCAC (TT-core Brachyury site necessary for notochord activity) sequence elements found in the *Ci-Fkbp9* CRM.  Abbreviations: bp: base pairs, occ.: occupancy, noto.: notochord, Bra.: Brachyury, clust.: cluster, act.: activity. | | | | | | | |
