# Supplementary material for: Brachyury, Foxa2 and the cis-Regulatory Origins of the Notochord
Source: PLoS Genet. 2015 Dec 18;11(12):e1005730. doi: 10.1371/journal.pgen.1005730 (PMC4684326; doi:10.1371/journal.pgen.1005730)
Supplement: S5 Table — (DOCX) [file pgen.1005730.s009.docx]

| **Table S5: Primers utilized for the PCR amplification of the most relevant constructs used for CRM characterization** | | |
| --- | --- | --- |
| **Primer/Construct name** | **Primer sequence (5' to 3')** | **Restriction site(s) used** |
| **Ci-CRM9** | | |
| 1-1751 | N/A | Eco01019I/NcoI |
| 1752-2181  (431 bp) | N/A | Eco01019I/Xba |
| 1-911 | N/A | SnaBI/Nco |
| 382-911 F  (530 bp) | AtctagaCCTGGTATATGCTGGAATAATGAAC | XbaI/NcoI^b^ |
| 600-911 F  (312 bp) | AtctagaGGAAGTGGGTGACCGTTGAATC | XbaI/NcoI^b^ |
| 382-911 B2 mutant F | GAAGAGTCAAagaCGAACATTGG | XbaI/NcoI^b^ |
| 382-911 B2 mutant R | CCAATGTTCGtctTTGACTCTTC |  |
| 382-911 B3 mutant F | TCTCTtctCTAAGTGCCTTATAAG | XbaI/NcoI^b^ |
| 382-911 B3 mutant R | CTTATAAGGCACTTAGagaAGAGA |  |
| 382-911 B2 CTAM mutant F | CTCTCTCACagccGTGCCTTA | XbaI/NcoI^b^ |
| 382-911 B2 CTAM mutant R | TAAGGCACggctGTGAGAGAG |  |
| **Ci-CRM24** | | |
| 1-1009 | N/A | PmeI/XbaI |
| 1009-2123  (1115 bp) | N/A | PmeI/NcoI |
| 1-391 | N/A | BsaAI/XbaI |
| 389-1009 (621 bp) | N/A | BsaAI/NcoI |
| 1-139 | CtctagaCGCAACAGCGGTGGCTTTC | XbaI/NcoI^b^ |
| 1-139 F1 mutant F | CAGGTTTgggcagTTCCTCG | XbaI/NcoI^b^ |
| 1-139 F1 mutant R | CGAGGAActgcccAAACCTG |  |
| 1-139 B4 mutant F | GTTTATTTACTTCCTCGTtaaTAAATAAACAAATAC | XbaI/NcoI^b^ |
| 1-139 B4 mutant R | GTATTTGTTTATTTAttaACGAGGAAGTAAATAAAC |  |
| 1-139 F1-B4 mutant top #1 | ctagaAATCTacaTTACTTGCGAAGtgtACA | XbaI/NcoI |
|  | TCACCCGtctTGACGGATCTCAAACCAGG |  |
| 1-139 F1-B4 mutant top #2 | TTTATTTACTTCCTCGTtaaTAAATAAA |  |
|  | CAAATACAGATCGCAAGGCTTTGTCA |  |
| 1-139 F1-B4 mutant top #3 | TATTGAACTCTGAAAGCCACCGCTGTTGCGgcatg |  |
| 1-139 F1-B4 mutant bottom #1 | GTTTGAGATCCGTCAagaCGGGTGA |  |
|  | TGTacaCTTCGCAAGTAAtgtAGATTt |  |
| 1-139 F1-B4 mutant bottom #2 | AAAGCCTTGCGATCTGTATTTGTTTAT |  |
|  | TTAttaACGAGGAAGTAAATAAACCTG |  |
| 1-139 F1-B4 mutant bottom #3 | cCGCAACAGCGGTGGCTTTCAGAGTTCAATATGAC |  |
| 1-139 F1/B4 mutant F | GTTTgggcagTTCCTCGTtaaTAAATAAAC | XbaI/NcoI^b^ |
| 1-139 F1/B4 mutant R | GTTTATTTAttaACGAGGAActgcccAAAC |  |
| 1-139 F1-F3/B4 mut F  (Template: F1 mut) | CTCGTGTtgcccgcccCAAATAC | XbaI/NcoI^b^ |
| 1-139 F1-F3/B4 mut R  (Template: F1 mut) | GTATTTGgggcgggcaACACGAG |  |
| **Ci-CRM26** | | |
| 1-1387 | N/A | EcoNI/XhoI |
| 1393-1868  (476 bp) | N/A | EcoNI/NcoI |
| 1-599 | N/A | PacI/XhoI |
| 610-1392 (782bp) | N/A | PacI/NcoI |
| 749-1080 (332 bp) | TTccatggTTTCGTGCCGCTGGTCTCG | XhoI/NcoI^b^ |
| 871-1068 (198 bp) | TTccatggTCTCGTTTTAGAGGCAGCGC | XbaI/NcoI^b^ |
| 922-1068 (147 bp) | TtctagaTACTTAAACGTCAACTGCTTACTTAAAC | XbaI/Nco^b^ |
| 906-992 F (87 bp) | TtctagaTTAGCTCGTCAACTGCTTACTTAAAC | XbaI/NcoI |
| 906-992 R (87bp) | TTccatggTCGCCGTGTGTCTAGTCACC |  |
| 906-992 E1 mut | ActcgagTAGCTCGTCAtacGCTTACTTAAAC | XhoI/NcoI^b^ |
| **Ci-CRM66** | | |
| 1-814 F | TtctagaTGcACGGTTAGGTTAAAGGCC | XbaI/XhoI |
| 1-814 R | ActcgagCAATACGACGTTTACCGTTCAATAC |  |
| 779-1091 F  (331 bp) | GtctagaGAAATCTAAATGTATTGAACGGTAAACG | XbaI/NcoI |
| 779-1091 R  (331 bp) | AAccatggCAATACTACAATGTTTATATTACATCTGTG |  |
| 779-1026 (248 bp) | TTccatggCCTAGATTGACCTGACTTAATCAAC | XbaI/NcoI^b^ |
| 779-1091 B2/F2 mutant F | CAATCTAGGTCAagaACACACACAC | XbaI/NcoI^b^ |
| 779-1091 B2/F2 mutant R1 | GTGTGTGTGTtctTGACCTAGATTG |  |
| 779-1091 B2/F2 mutant R2 | GAccatggCAATACTACAATGgggcgATTACATCTG |  |
| 839-1091 (253 bp) | GTtctagaCTCTACAGTATACGGAGAGTCC | XbaI/NcoI^b^ |
| 839-1091 B2 mutant F | CAATCTAGGTCAagaACACACACAC | XbaI/NcoI^b^ |
| 839-1091 B2 mutant R | GTGTGTGTGTtctTGACCTAGATTG |  |
| 839-1026 (187 bp) (Template: 839-1091) | (779-1026 primer, see above) | XbaI/NcoI^b^ |
| 839-1091 LSM1 F | GTCACACcacacacaACACTGTA | XbaI/NcoI^b^ |
| 839-1091 LSM1 R | TACAGTGTtgtgtgtgGTGTGAC |  |
| 839-1091 LSM2 F | CACACACAacgcagcgATGTATAT | XbaI/NcoI^b^ |
| 839-1091 LSM2 R | ATATACATcgctgcgtTGTGTGTG |  |
| 839-1091 LSM3 F | CACACTGgcatacgcTACACAAC | XbaI/NcoI^b^ |
| 839-1091 LSM3 R | GTTGTGTAgcgtatgcCAGTGTG |  |
| 839-1091 LSM4 F | GTATGTATcgcataccCACAGATG | XbaI/NcoI^b^ |
| 839-1091 LSM4 R | CATCTGTGggtatgcgATACATAC |  |
| 839-1091 LSM5 F | GTTTATATTACcgagtatgTGTGTATATAC | XbaI/NcoI^b^ |
| 839-1091 LSM5 R | GTATATACACAcatactcgGTAATATAAAC |  |
| 839-1091 LSM6 | AAccatggCAATACTACAATGTTTATcggcacTCTG | XbaI/NcoI^b^ |
| 839-1091 LSM7 | AAccatgggccgcagtactTGTTTATATTACATC | XbaI/NcoI^b^ |
| 839-1091 (AC)_2_ mutant F | CTAGGTCACACttttACACACACTG | XbaI/NcoI |
| 839-1091 (AC)_2_ mutant R | CAGTGTGTGTaaaaGTGTGACCTAG |  |
| 839-1091 (AC)_6_ mutant F | GGTCACACttttttttttttTGTATGTATATAC | XbaI/NcoI |
| 839-1091 (AC)_6_ mutant R | GTATATACATACAaaaaaaaaaaaaGTGTGACC |  |
| **Ci-CRM70** | | |
| 1-1769 | N/A | BsrGI/NcoI |
| 202-1557 F  (1356 bp) | AtctagaAGAAATGTTTAATATTGCCCAAGTCCC | XbaI/BamHI |
| 202-1557 R  (1356 bp) | TggatccTCCGGACGACGCCATTCC |  |
| 1557-1769  (213 bp) | AtctagaAAATTGATGCAAGAAGTTTTATATTCGCG | XbaI/NcoI^b^ |
| 1633-1760 F  (128 bp) | AtctagaGAATTTACTCGATATTTGTATTGTTACGTC | XbaI/NcoI |
| 1633-1760 R  (128 bp) | TTccatggTCACATATATGCGTTGTGACGTCATATAC |  |
| 1682-1760 (79bp) | AtctagaGAATTTACTCGATATTTGTATTGTTACGTC | XbaI/NcoI^b^ |
| 1633-1760 M3-1 mutant F | CTTATCCACAaccaGGTTCAACC | XbaI/NcoI^b^ |
| 1633-1760 M3-1 mutant R | GGTTGAACCtggtTGTGGATAAG |  |
| **Ci-CRM76** | | |
| 30-618 F (682 bp) | TtctagaTGGCAGTCTCATACATTCTAATCTGG | XbaI/BamHI |
| 30-618 R (682 bp) | AggatccACAGCATAGTCACAACGGCTTTC |  |
| 186-618 (433 bp) | TtctagaTGCCAGGTATGTCTAAATTCAACATG | XbaI/BamHI^b^ |
| 306-618 (313 bp) | TtctagaTAGTGACTCAAGTTTCAAAGTTATGTTAAG | XbaI/BamHI^b^ |
| 306-618 B1 mutant F | CGTGAATTGAAATGTataTTAACTTAACCTAG | XbaI/BamHI^b^ |
| 306-618 B1 mutant R | CTAGGTTAAGTTAAtatACATTTCAATTCACG |  |
| 441-618 (178 bp) | AtctagaCCAGTAATGAGTTTCGTACAGAATAC | XbaI/BamHI^b^ |
| 441-618 M2-1, M1-4, M3-1 mutant F | GCCGTGAAccaAAATGTGTaccAACTTAAC | XbaI/BamHI^b^ |
| 441-618 M2-1, M1-4, M3-1 mutant R | GTTAAGTTggtACACATTTtggTTCACGGC |  |
| 441-618 M2-1, M1-4, M3-1 mutant R2 | AggatccACAGCATAGTCACggtGGCTTTC |  |
| 497-618 (122 bp) | AtctagaCTTAACCTAGATTGAATGCATTCCG | XbaI/BamHI^b^ |
| 525-618 (94 bp) | AtctagaCGAGTACAAATGCCTTTCTGATTTG | XbaI/BamHI^b^ |
| 186-618 M1-5 mutant F | GTGTGTTAACTTggtCTAGATTGAATGC | XbaI/BamHI^b^ |
| 186-618 M1-5 mutant R | GCATTCAATCTAGaccAAGTTAACACAC |  |
| 186-618 M2-2 mutant F | CTTAACCTAGAccaAATGCATTCCG | XbaI/BamHI^b^ |
| 186-618 M2-2 mutant R | CGGAATGCATTtggTCTAGGTTAAG |  |
| 186-618 M1-5/M2-2 mutant F | GTTAACTTggtCTAGAccaAATGCATTC | XbaI/BamHI^b^ |
| 186-618 M1-5/M2-2 mutant R | GAATGCATTtggTCTAGaccAAGTTAAC |  |
| 186-618 M2-1 ori rev F | GTTAACTTAACCTAGttcaatTGCATTCC | XbaI/BamHI^b^ |
| 186-618 M2-1 ori rev R | GGAATGCAattgaaCTAGGTTAAGTTAAC |  |
| 186-618 order rev F | GTTAACTattgaaCTAGtaacTGCATTCC | XbaI/BamHI^b^ |
| 186-618 order rev R | GGAATGCAgttaCTAGttcaatAGTTAAC |  |
| 186-618 insertion F | GTTAACTTAACCTAGcatcATTGAATGC | XbaI/BamHI^b^ |
| 186-618 insertion R | GCATTCAATgatgCTAGGTTAAGTTAAC |  |
| **Ci-CRM86** | | |
| 1241-1698 F  (458 bp) | AtctagaCGACTTACCCCAACCTACTATATTC | XbaI/BamHI |
| 1241-1698 R  (458 bp) | AggatccGATTACAGAACCGAATCGTGTTCG |  |
| 1446-1698  (253 bp) | TtctagaGTACCAGgCACACACGAGATATC | XbaI/BamHI^b^ |
| 1241-1468  (228 bp) | AggatccGATATCTCGTGTGTGcCTGGTAC | XbaI/BamHI^b^ |
| 1241-1468 F1 mutant F | GAAAGTTTTgggcAgCGAAATAAC | XbaI/BamHI^b^ |
| 1241-1468 F1 mutant R | GTTATTTCGcTgcccAAAACTTTC |  |
| 1241-1468 F2 mutant F | GATGTTAtGGtCCcACCGGTTT | XbaI/BamHI^b^ |
| 1241-1468 F2 mutant R | AAACCGGTgGGaCCaTAACATC |  |
| 1241-1468 B2 mutant F | CTCTAAATATAAtatCTACAGATGTTATATTTAC | XbaI/BamHI^b^ |
| 1241-1468 B2 mutant R | GTAAATATAACATCTGTAGataTTATATTTAGAG |  |
| 1241-1468 B2 CTAM mutant F | GTGACTCTAAATATAACACagcaAGATG | XbaI/BamHI^b^ |
| 1241-1468 B2 CTAM mutant F | CATCTtgctGTGTTATATTTAGAGTCAC |  |
| **Ci-CRM90** | | |
| 573-885 F (313 bp) | TACAtctagaCTAGCCGGTGGATAGGCCGGGCC | XhoI/XbaI |
| 573-885 R (313bp) | ACTActcgagGTTTGGCTTTCGGGCGGCCACC |  |
| 573-885 B1 mutant F | ACTAGATGGagtACAGCCAAG | XhoI/XbaI^b^ |
| 573-885 B1 mutant R | CTTGGCTGTactCCATCTAGT |  |
| 590-885 (296 bp) | TTgcatgcCCGGGCCAAGTGCCTGG | XhoI/SphI^b^ |
| 573-817 (245 bp) | TctcgagGGTGCCATCTAGTACCTGG | XhoI/XbaI |
| 573-817 S/K1 mutant | TAgcatgcACTAGCCttaGGATAGGCCG | XhoI/SphI^b^ |
| **Ci-CRM96** | | |
| 959-1101; 1200-1307 F (251 bp) | GTCActcgagCAGTGGACAAGTTTGTGAAGG | XhoI/XbaI |
| 959-1101; 1200-1307 R (251 bp) | TTCGtctagaGTAAGCGAGCACGAGGTGTGTG |  |
| 1228-1307 (80 bp) | ActcgagCTTGTTTATTCAACCATGGCGG | XhoI/XbaI^b^ |
| 1237-1307 (71 bp) | TctcgagTCAACCATGGCGGGGCAAC | XbaI/XhoI^b^ |
| 1124-1307 F2 mutant F | ACGCATGTAACTTGgggcgTCAACCATG | XhoI/NcoI^b^ |
| 1124-1307 F2 mutant R | CATGGTTGAcgcccCAAGTTACATGCGT |  |
| 1124-1307 B3 mutant F | CAGTTATTATAAagaCGGTGGTCTG | XhoI/XbaI^b^ |
| 1124-1307 B3 mutant R | CAGACCACCGtctTTATAATAACTG |  |
| 1124-1307 B4 mutant F | AtctagaGTAAGCGAGCACGAGGTtctTG | XhoI/XbaI^b^ |
| **Ci-CRM99** | | |
| 1-749 | N/A | NsiI/XhoI |
| 754-1563 (810 bp) | N/A | NsiI/NcoI |
| 1-749 F3 mutant F | TAGTTTGTcgcccCAGAACTG | XhoI/NcoI^b^ |
| 1-749 F3 mutant R | CAGTTCTGgggcgACAAACTA |  |
| 1-749 B8 mutant F | GATATACTGATGTagaCCATGTACGCTG | XhoI/NcoI^b^ |
| 1-749 B8 mutant R | CAGCGTACATGGtctACATCAGTATATC |  |
| 441-755 F (228 bp) | AAgcatgcAGCTTTTTTGGTAAGCTATTTGCTTGTG | XhoI/SphI |
| 441-755 R (228 bp) | ActcgagACATGCATACATCAAAACACATTGTATAAAG |  |
| 205-749 (547 bp) | N/A | PmlI/NcoI |
| 1-749 B4 mutant F | TGTCAACACTGAagtTTAGTTGTTG | XhoI/NcoI^b^ |
| 1-749 B4 mutant R | CAACAACTAAactTCAGTGTTGACA |  |
| 1-749 B4 CTAM mutant F | CACTGACACggctTTGTTGATTAC | XbaI/NcoI^b^ |
| 1-749 B4 CTAM mutant R | GTAATCAACAAagccGTGTCAGTG |  |
| **Ci-CRM109** | | |
| 1-860 | N/A | BstAPI/NcoI |
| 864-1585 (722 bp) | N/A | BstAPI/XbaI |
| 939-1136 F  (198 bp) | CtctagaCAAACATCTTGCACCTAAGTGCTG | XbaI/NcoI |
| 939-1136 R  (198 bp) | AAccatggCGTAATTATAACCTGGCACGAGATG |  |
| 939-1136 B1 mutant | AtctagaCAAACATCTTGagaCTAAGTGCTG | XbaI/NcoI^b^ |
| 939-1136 B1 CTAM mutant | AtctagaCAAACATCTTGCACagccGTGCTG | XbaI/NcoI^b^ |
| **Ci-CRM112** | | |
| 594-738 F (145 bp) | GTCAggatccGAATGTTACGCGTTAGAAAGTCG | PstI/BamHI |
| 594-738 R (145 bp) | CAGTctgcagCAACGATAGCGCGAGAGAGAAC |  |
| 594-703 (110 bp) | CTccatggGTGCAGCGAAACTCATGCGG | BamHI/NcoI^b^ |
| 594-687 (94 bp) | TTccatggGCGGTCAGAGCGAGACTG | BamHI/NcoI^b^ |
| 594-674 (81 bp) | CAccatggGACTGTTTGTCTAATTTCGCATCAG | BamHI/NcoI^b^ |
| 594-650 (57 bp) | CCAAccatggGTGCCGAACCGAAGAGTATTTG | BamHI/NcoI^b^ |
| 594-687 HD mutant | TAccatggGCGGTCAGAGCGAGACTGTTTGTCgtcgTTC | BamHI/NcoI^b^ |
| 594-687 F2 mutant F | CAccatggGCGGTCAGAGCGAGACTtgggtTCTAATTTC | BamHI/NcoI^b^ |
| 594-687 AP1 mutant | TAccatggGCGactgGAGCGAGACTG | BamHI/NcoI^b^ |
| 594-687 HD/F2 mutant | CAccatggGCGGTCAGAGCGAGACTtgggtTCgtcgTTC | BamHI/NcoI^b^ |
| 594-687 F2/AP1 mutant | CAccatggGCGactgGAGCGAGACTtgggtTCTAATTTC | BamHI/NcoI^b^ |
| ***C6ST*** | | |
| 1060-1434 F  (375 bp) | CggatccTTACCCCACAGTGATATAACTTCTTTATTTG | BamHI/NcoI |
| 1060-1434 R  (375 bp) | CATGccatggCCCTCGCATTCCACATTCTCG |  |
| 1271-1434  (164 bp) | N/A | XbaI |
| 1271-1288; 1319-1434 F (134 bp) | GGTTCCTAGGGTAAATACCGTCTGATTATTTC | XbaI/NcoI^b^ |
| 1271-1288; 1319-1434 R (134 bp) | GGTTCCTAGGATCTAGGATTCTCTAGAG |  |
| 1271-1353 F  (83 bp) | CggatccTAGAGAATCCTAGATTGCAAGGCCTTTTG | BamHI/NcoI |
| 1271-1353 R  (83 bp) | ACGTCATccatggCAAGTATATCTGCGAGAAATAATCAG |  |
| 1354-1434 (81 bp) | CggatccTTACATTCCTAAGAGTAAACATAGAGTTTTCG | BamHI/NcoI^b^ |
| 1271-1434 M1-2 mutant F | TCTCGCAGATACTTGggcCATTCCTAAGAGTAA | XbaI/NcoI |
| 1271-1434 M1-2 mutant R | TTACTCTTAGGAATGgccCAAGTATCTGCGAGA |  |
| ***FKBP9*** | | |
| 750-1061 F  (312 bp) | CTTGtctagaGTATATGATGTCTATGTTTTATAG | XbaI/NcoI |
| 750-1061 R  (312 bp) | CAATccatggCAGCATGTCTAGCTTTTGTGC |  |
| 901-1061 (161 bp) | TGGCtctagaGGGGACATATATTGAATTAC | XbaI/NcoI^b^ |
| 973-1061 F (89 bp) | AGctcgagCTTATCCACCAATTGCTTTCCA | XhoI/XbaI |
| 973-1061 R (89 bp) | GAtctagaCAGCATGTCTAGCTTTTG |  |
| 973-1061 E1/B3 mutant F1 | CTTATCCACagatcaCTTTCCAGCA | XhoI/XbaI^b^ |
| 973-1061 E1/B3 mutant R1 | TGCTGGAAAGtgatctGTGGATAAG |  |
| 973-1061 E1/B3 mutant R2 | GAtctagaCAGCATGTCTAGCTTTTagaCAATAAG |  |
| 973-1027 (55 bp) | GAtctagaAAGTAACACGTGACTTCAACATTTG | XhoI/XbaI |
| 973-1061 B2 mutant | GAtctagaCAGCATGTCTAGCTTTTGTGCAATAAGGCagaAAAAG | XhoI/XbaI |
| **Predictions** | | |
| Noto2 F | CctcgagCAAAGTGAAAGGCAAGCATTTCCG | XhoI/NcoI |
| Noto2 R | CTccatggGAGCATATGGTTCACCCACAG |  |
| ^a^Artificial restriction sites are in lowercase, while mutated nucleotides are lowercase and underlined. | | |
| ^b^Corresponding primer in the opposite direction is specific to the reporter plasmid or to a previously made construct with the same boundaries. | | |
| N/A: construct was prepared by restriction digestion using endogenous restriction sites. | | |
